# Clinical Efficacy of Mobile App–Based, Self-Directed Pulmonary Rehabilitation for Patients With Chronic Obstructive Pulmonary Disease: Systematic Review and Meta-Analysis

**DOI:** 10.2196/41753

**Published:** 2024-01-04

**Authors:** Chiwook Chung, Jong Won Lee, Sei Won Lee, Min-Woo Jo

**Affiliations:** 1 Department of Pulmonary and Critical Care Medicine, Asan Medical Center, University of Ulsan College of Medicine, Seoul, Republic of Korea; 2 Department of Pulmonary and Critical Care Medicine, Gangneung Asan Hospital, University of Ulsan College of Medicine, Gangneung, Republic of Korea; 3 Division of Breast Surgery, Department of Surgery, Asan Medical Center, University of Ulsan College of Medicine, Seoul, Republic of Korea; 4 Department of Preventive Medicine, Asan Medical Center, University of Ulsan College of Medicine, Seoul, Republic of Korea

**Keywords:** pulmonary rehabilitation, COPD, chronic obstructive pulmonary disease, mobile application, mobile app, 6MWD, 6-minute walk test distance, CAT, COPD Assessment Test, mMRC, modified Medical Research Council, SGRQ, St. George Respiratory Questionnaire, exacerbation, rehabilitation, mHealth, mobile health, clinical efficacy, PRISMA, mobile phone

## Abstract

**Background:**

Pulmonary rehabilitation is well known to improve clinical symptoms (including dyspnea), quality of life, and exercise capacity in patients with chronic obstructive pulmonary disease (COPD). However, researchers have reported difficulties in practicing center-based pulmonary rehabilitation. Recently, mobile app–based pulmonary rehabilitation has become available in clinical practice. We investigated the clinical outcomes of mobile app–based pulmonary rehabilitation in patients with COPD.

**Objective:**

The objective of our study was to evaluate the clinical efficacy of mobile app–based pulmonary rehabilitation versus conventional center-based pulmonary rehabilitation for patients with COPD, using a systematic review and meta-analysis.

**Methods:**

A systematic search of the literature published between January 2007 and June 2023 was performed, using the PubMed, Embase, Cochrane, and CINAHL databases to identify relevant randomized controlled trials involving patients with COPD. Pulmonary rehabilitation programs needed to provide an exercise program on a smartphone app. Study outcomes, including exercise capacity, symptom scores, quality of life, and hospitalization, were evaluated. The meta-analysis evaluated mean differences in 6-minute walk test distances (6MWDs), COPD Assessment Test (CAT) scores, modified Medical Research Council (mMRC) dyspnea scale scores, St. George Respiratory Questionnaire (SGRQ) scores, and risk ratios for hospitalization resulting from disease exacerbation.

**Results:**

Of the 1173 screened studies, 10 were included in the systematic review and 9 were included in the meta-analysis. Further, 6 studies were multicenter studies. There were a total of 1050 participants, and most were aged ≥65 years. There were discrepancies in the baseline participant characteristics, smartphone apps, interventions, and study outcomes among the included studies. In the meta-analysis, 5 studies assessed 6MWDs (mean difference 9.52, 95% CI −3.05 to 22.08 m), 6 studies assessed CAT scores (mean difference −1.29, 95% CI −2.39 to −0.20), 3 studies assessed mMRC dyspnea scale scores (mean difference −0.08, 95% CI −0.29 to 0.13), 2 studies assessed SGRQ scores (mean difference −3.62, 95% CI −9.62 to 2.38), and 3 studies assessed hospitalization resulting from disease exacerbation (risk ratio 0.65, 95% CI 0.27-1.53). These clinical parameters generally favored mobile app–based pulmonary rehabilitation; however, a statistically significant difference was noted only for the CAT scores (*P*=.02).

**Conclusions:**

Despite some discrepancies in the baseline participant characteristics and interventions among studies, mobile app–based pulmonary rehabilitation resulted in favorable exercise capacity, symptom score, quality of life, and hospitalization outcomes when compared with conventional pulmonary rehabilitation. In the meta-analysis, the CAT scores of the mobile app–based pulmonary rehabilitation group were significantly lower than those of the control group (*P*=.02). In real-world practice, mobile app–based pulmonary rehabilitation can be a useful treatment option when conventional center-based pulmonary rehabilitation is not feasible.

## Introduction

Chronic obstructive pulmonary disease (COPD) is characterized by persistent respiratory symptoms and airflow limitation, which are usually caused by exposure to noxious gases or particles [1]. Recently, the prevalence of COPD has increased, making it a leading cause of morbidity and mortality worldwide [2,3]. Approximately 3,500,000 people experience COPD, and it is the third leading cause of disability-adjusted life years (1305 disability-adjusted life years per 100,000 population, 6.21% of total noncommunicable diseases disability-adjusted life years) in South Korea [4,5]. COPD has various extrapulmonary features and might be a systemic disease rather than a disease that only affects the airway [6]. Various clinical information is relevant to the mortality of patients with COPD, including information on physical activity, disability, lung function, long‐term oxygen therapy, BMI, quality of life, depressive symptoms, marital status, comorbidity, and hospitalization [7-9]. Additionally, the BODE (BMI, Airflow Obstruction, Dyspnea, and Exercise Capacity) index, which includes BMI, airflow obstruction as assessed by the forced expiratory volume in 1 second (FEV_1_), dyspnea as assessed by the modified Medical Research Council (mMRC) dyspnea scale, and exercise capacity as assessed by the 6-minute walk test distance (6MWD), is well known to predict mortality in patients with COPD [10,11].

Pulmonary rehabilitation is a comprehensive intervention for improving the physical and psychological conditions of people with chronic respiratory diseases through exercise training, education, and behavior modification [12]. Pulmonary rehabilitation has been shown to improve dyspnea, quality of life, and exercise capacity in patients with COPD [1,12-14]. Furthermore, patients with chronic respiratory diseases have decreased respiratory muscle mass and strength, which are accompanied by decreased respiratory function. In this population, pulmonary rehabilitation with exercise training is the only way to improve respiratory function [15]. The pulmonary rehabilitation programs used in previous landmark studies were composed of exercise training that was performed 30 to 45 minutes per day, 3 to 5 days per week, for at least 8 to 12 weeks [16,17]. However, researchers reported difficulties in practicing center-based pulmonary rehabilitation, including a lack of facilities; low health insurance coverage; a lack of awareness among physicians; and a lack of motivation, transport barriers, and low levels of social support among patients [4,18,19]. Thus, alternatives to center-based pulmonary rehabilitation are desperately needed [20]. Recently, the demand for telerehabilitation in pulmonary rehabilitation is increasing, owing to advances in telemedicine and challenges with face-to-face rehabilitation during the COVID-19 pandemic [20-22]. Among telerehabilitation modalities, mobile app–based pulmonary rehabilitation has been used in clinical trials; however, the clinical evidence for mobile app–based pulmonary rehabilitation from these studies has been inconclusive due to the heterogeneity in participants, study designs, and formats of apps [23-32]. Furthermore, previous systematic reviews focused on telerehabilitation [20], home telemonitoring [33], or patient support apps [34]. Therefore, we aimed to compare the clinical outcomes of mobile app–based, self-directed pulmonary rehabilitation programs (ie, those without telemonitoring but with exercise programs) in patients with COPD to those of conventional pulmonary rehabilitation because exercise programs are key components of pulmonary rehabilitation that improve chronic respiratory diseases and health-enhancing behaviors [12].

## Methods

### Data Sources and Literature Search

Literature searches were performed by using the PubMed, Embase, Cochrane, and CINAHL databases. The searches were conducted for literature published since 2007 because the iPhone (Apple Inc) and Android (Google LLC) smartphones were released in June 2007 and September 2008, respectively. The databases were searched for literature published up to June 30, 2023. Only full-text studies written in English were included. The search strategy was based on a PICOTS-SD (population, intervention, comparison, outcomes, time, setting, and study design) list (Multimedia Appendix 1). Briefly, the search algorithm focused on keywords related to “chronic pulmonary disease,” “mobile application,” and various clinical outcomes. If needed, authors were contacted for further information.

### Eligibility Criteria and Study Selection

Each study was reviewed by 2 authors (CC and MWJ) independently according to the inclusion and exclusion criteria. The inclusion and exclusion criteria are presented in Table 1. The screening of titles and abstracts and the subsequent full-text review were performed by 2 authors (CC and MWJ) independently. Disagreements during the selection process were resolved through a discussion between 3 authors (CC, MWJ, and SWL).

**Table 1.** Inclusion and exclusion criteria.

	Inclusion criteria	Exclusion criteria
Article type	Full-text articles	Abstracts, conference posters, and grey literature
Language	English	Not English
Study design	Randomized controlled trials	Nonrandomized trials, literature reviews, and protocols
Participants’ age	Adults	Adolescents
Disease	COPD^a^	Other respiratory diseases, such as asthma
Smartphone app	Conventional or newly developed smartphone apps	Cellular phones
Intervention	Pulmonary rehabilitation, including exercise programs, provided by a smartphone app	Self-management programs, step counters, peak flow meters, etc
Control	Conventional pulmonary rehabilitation, including exercise programs (center-based rehabilitation or education)	N/A^b^
Study outcome	At least 1 of the following outcomes: 6-minute walk test distance, COPD Assessment Test score, modified Medical Research Council dyspnea scale score, St. George Respiratory Questionnaire score, and hospitalization resulting from disease exacerbation	N/A

a COPD: chronic obstructive pulmonary disease.

b N/A: not applicable.

### Data Collection and Risk of Bias Assessment

Two authors (CC and MWJ) independently collected data regarding (1) general information about the study (authors, year, country, and study setting), (2) descriptions of study arms (number, sex, and age of participants), (3) characteristics of interventions, (4) inclusion and exclusion criteria, and (5) results for outcomes; they also double-checked these data. Two authors (CC and MWJ) independently assessed the risk of bias in the included studies. Discrepancies were resolved in discussions with the third author (SWL).

### Study Outcomes

In the meta-analysis, study outcomes, including exercise capacity, symptom scores, quality of life, and hospitalization, were assessed. Exercise capacity was measured by using 6MWDs. The symptom scores were measured by using the COPD Assessment Test (CAT) and the mMRC dyspnea scale. Quality of life was measured by using the St. George Respiratory Questionnaire (SGRQ). *Hospitalization* was defined as hospitalizations resulting from disease exacerbation. The primary time points for the analysis were baseline and the end of the intervention.

### Statistical Analysis

The continuous variables included the 6MWD, CAT score, and SGRQ score. The mMRC dyspnea scale score was a categorical variable, and it was calculated as a continuous value. Hospitalization resulting from disease exacerbation was a dichotomous variable. The variables at the time of follow-up were compared between groups. The mean differences and risk ratios between the intervention group and the control group were calculated, along with 95% CIs. The chi-square test and the *I*^2^ statistic were used to assess statistical heterogeneity. If *I*^2^ was <50%, the fixed effect model was used. Publication bias was visually assessed by using a funnel plot analysis because the limited number of studies with results for each outcome prevented us from performing the Egger test. The meta-analysis was performed by using Review Manager (RevMan) version 5.4 (The Cochrane Collaboration).

### Ethical Considerations

This study complied with the Declaration of Helsinki, and all methods were performed in accordance with the relevant guidelines.

## Results

### Study Selection

**Figure 1.**
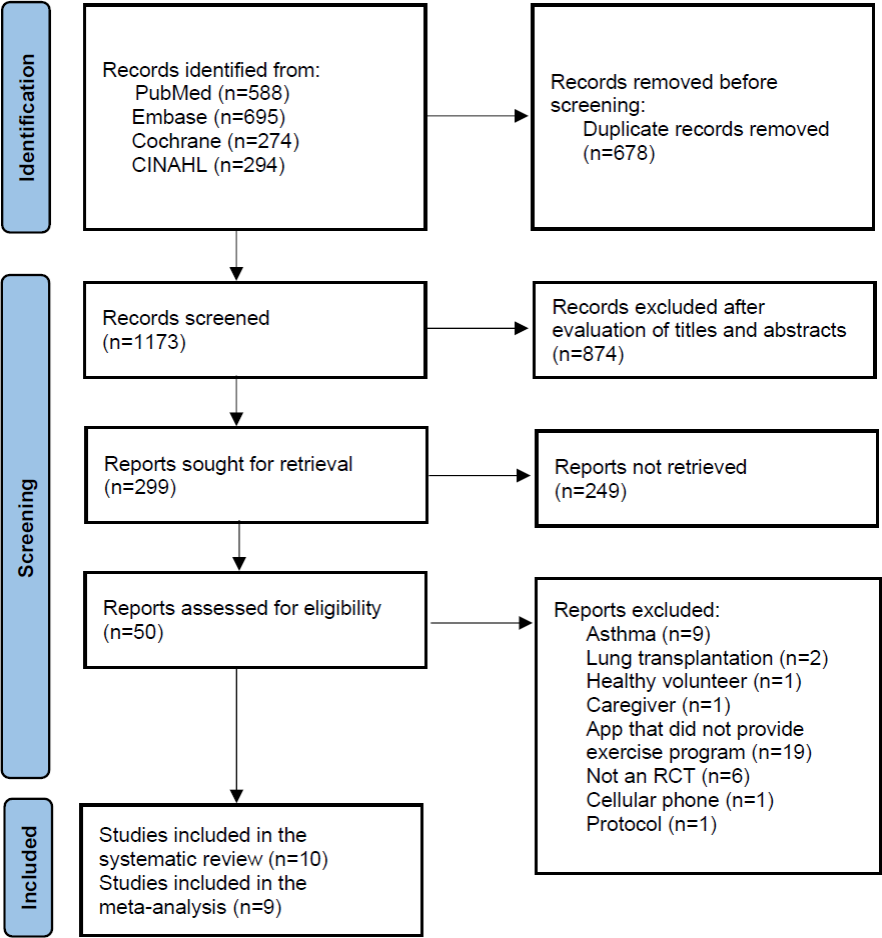
PRISMA (Preferred Reporting Items for Systematic Reviews and Meta-Analyses) diagram of the literature search and selection process. Studies were identified via databases and registers. RCT: randomized controlled trial.

An initial literature search identified a total of 1851 articles from the PubMed, Embase, Cochrane, and CINAHL databases; thereafter, 1173 articles remained after duplicates were removed. After evaluating titles and abstracts, 299 articles remained eligible for a full-text review. The full-text review was performed according to the criteria mentioned in the *Eligibility Criteria and Study Selection* section, and 10 articles were finally included in the systematic review [23-32]. Notably, 1 study was excluded from the meta-analysis because exercise capacity was evaluated by using the incremental shuttle walk test (ISWT) instead of 6MWDs [32]. Therefore, 9 studies were included in the meta-analysis [23-31] (Figure 1).

### Characteristics of Included Studies

Characteristics of studies are described in Table 2. Studies were published after 2014, with almost half of them (4/10, 40%) published in 2020 [24,26,28,29]. Further, 6 studies were multicenter studies [24,25,27,29-31], and 3 studies enrolled fewer than 50 participants; the largest number of participants was 343 [25,28,29,32]. There were 1050 total participants, who were generally aged ≥65 years. More male participants were enrolled than female participants, and Wang et al [32] enrolled only male participants. In the study by North et al [28], participants were recruited after hospital admission with an acute exacerbation. In the studies by Vorrink et al [31] and Wang et al [32], participants were recruited after pulmonary rehabilitation. Kwon et al [27] recruited 2 groups of participants in the intervention arm, comprising the fixed regimen group and the fixed-interactive regimen group, according to exercise programs. Various formats of mobile apps were used for the studies; 2 studies in the United Kingdom used myCOPD, a digital health care app approved by the National Health Service [24,28], and 1 study in China used WeChat (Tencent Holdings Ltd), a popular mobile messenger app in China [26]. The follow-up duration ranged between 3 weeks and 12 months [23,31].

**Table 2.** Characteristics of included studies.

Study (author, year)		Setting	Country	Sample size, n (%)		Age (y), mean (SD)		Mobile app	Follow-up duration
				Intervention	Control	Intervention	Control		
Barata et al [23], 2022		Single center	Romania	Male: 42 (72.4); female: 16 (27.6)	Male: 54 (75); female: 18 (25)	64.3 (4.3)	64.9 (5.7)	Pneumocontrol app (newly developed)	21 d
Crooks et al [24], 2020		Multicenter	United Kingdom	Male: 11 (37.9); female: 18 (62.1)	Male: 20 (64.5); female: 11 (35.5)	65.9 (7.3)	66.4 (7.0)	myCOPD	90 d
Demeyer et al [25], 2017		Multicenter	Belgium	Male: 111 (64.9); female: 60 (35.1)	Male: 108 (62.8); female: 64 (37.2)	66 (8)	67 (8)	Fitbug app and a project-tailored coaching app	12 wk
Jiang et al [26], 2020		Single center	China	Male: 44 (83); female: 9 (17)	Male: 43 (81.1); female: 10 (18.9)	70.9 (6.4)	71.8 (7.6)	WeChat official account based on social media	6 mo
Kwon et al [27], 2018		Multicenter	Republic of Korea	Male^a^: 23 (85.2); female^a^: 4 (14.8); male^b^: 26 (86.7); female^b^: 4 (13.3)	Male: 21 (75); female: 7 (25)	64 (8)^a^; 65 (7)^b^	64 (8)	efil breath (newly developed)	12 wk
North et al^c^ [28], 2020		Single center	United Kingdom	Male: 13 (65); female: 7 (35)	Male: 11 (52.4); female: 10 (47.6)	65.1 (6.3)	68.1 (7.4)	myCOPD	90 d
Park et al [29], 2020		Multicenter	Republic of Korea	Male: 19 (86.4); female: 3 (13.6)	Male: 14 (70); female: 6 (30)	70.5 (9.4)	65.1 (11.1)	COPD^d^ self-management program (newly developed)	6 mo
Spielmanns et al [30], 2023		Multicenter	Switzerland	Male: 17 (51.5); female: 16 (48.5)	Male: 17 (50); female: 17 (50)	66.1 (6.8)	62.7 (8.2)	Kaia COPD app (newly developed)	6 mo
Vorrink et al^e^ [31], 2016		Multicenter	Netherlands	Male: 42 (50); female: 42 (50)	Male: 36 (49.3); female: 37 (50.7)	62 (9)	63 (8)	Newly developed	12 mo
Wang et al^e^ [32], 2014		Single center	Taiwan	Male: 12 (100)	Male: 14 (100)	71.4 (1.9)	71.9 (2.7)	Newly developed	6 mo

a The fixed regimen group.

b The fixed-interactive regimen group.

c Participants were recruited after hospital admission with an acute exacerbation.

d COPD: chronic obstructive pulmonary disease.

e Participants were recruited after pulmonary rehabilitation.

The interventions in the studies are described in Table 3. Disease education and monitoring were provided in 5 studies [24,26,28-30,35], and the other 5 studies provided only exercise programs [23,25,27,31,32]. The level of exercise could be adjusted according to the participants’ exercise capacity in 5 studies [23,25,27,31,32]. In particular, Kwon et al [27] provided 2 kinds of exercise regimens, and walking distances were adjustable in both regimens. In cases of COPD exacerbation or poor compliance to pulmonary rehabilitation, participants could contact health care professionals in 7 studies [24-26,28-30,32,35]. Jiang et al [26] gave incentives to participants, that is, participants could obtain gifts at a mall by using acquired points.

**Table 3.** Interventions of included studies.

Authors	Exercise adjustment	Exercise monitoring	Disease education	Disease monitoring	Social support	Contact with health care professionals	Incentive
Barata et al [23]	✓	✓		✓			
Crooks et al [24]			✓	✓		✓	
Demeyer et al [25]	✓	✓				✓	
Jiang et al [26]		✓	✓	✓	✓	✓	✓
Kwon et al [27]	✓	✓					
North et al [28]			✓	✓		✓	
Park et al [29]		✓	✓	✓	✓	✓	
Spielmanns et al [30]		✓	✓	✓		✓	
Vorrink et al [31]	✓	✓					
Wang et al [32]	✓	✓				✓	

Most studies (7/10, 70%) included adult participants with physician-diagnosed COPD; diagnoses were made according to the GOLD (Global Initiative for Chronic Obstructive Lung Disease) criteria [1]. Some studies did not include participants with severe COPD as defined by the GOLD criteria [29,31], and others did not set limitations for disease severity. Generally, participants with recent acute exacerbations, participants undergoing long-term home oxygen therapy, or participants with other medical conditions that did not allow for physical exercise were excluded. In the study by North et al [28], participants were included after hospitalization with an acute exacerbation (Table S1 in Multimedia Appendix 2).

Participants were evaluated on various dimensions of outcomes, including exercise capacity, disease severity, quality of life questionnaires, and acute exacerbation. Wang et al [32] reported favorable exercise capacity and serum inflammatory biomarker outcomes; however, this study was excluded from the meta-analysis because exercise capacity was reported based on the ISWT and limb muscle strength. Crooks et al [24] and North et al [28] reported that inhaler technique improved in the intervention group, which was beneficial to disease control. Demeyer et al [25] reported that lung function did not improve during pulmonary rehabilitation in the intervention and control groups, and musculoskeletal events occurred more often in the intervention group. Barata et al [23] reported that the maximal inspiratory and expiratory pressures improved in the intervention group (Table S2 in Multimedia Appendix 2).

### Risk of Bias in Studies

**Figure 2.**
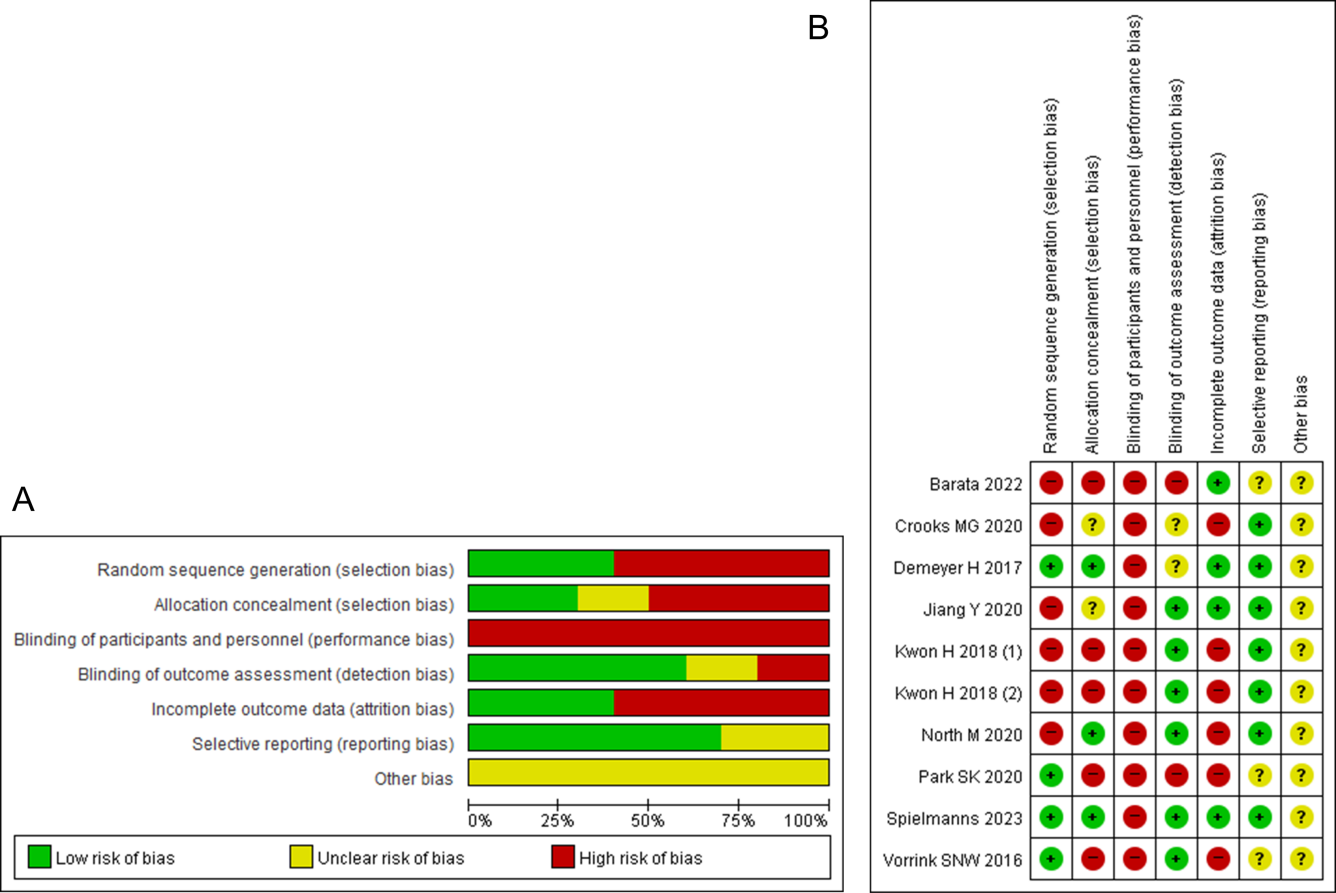
Risk of bias in the included studies [23-31]. A: Risk of bias graph. B: Risk of bias summary; “Kwon H 2018 (1)” denotes the fixed regimen group, and “Kwon H 2018 (2)” denotes the fixed-interactive regimen group.

The overall risk of bias in studies was considered low. However, the risk of performance bias was inevitably considered high in all studies because participant blinding was impossible, owing to the nature of the intervention (Figure 2). Funnel plots of comparisons showed fairly symmetrical distributions, which might mean less publication bias (Figure S1 in Multimedia Appendix 3).

### Meta-Analysis of Clinical Outcomes

**Figure 3.**
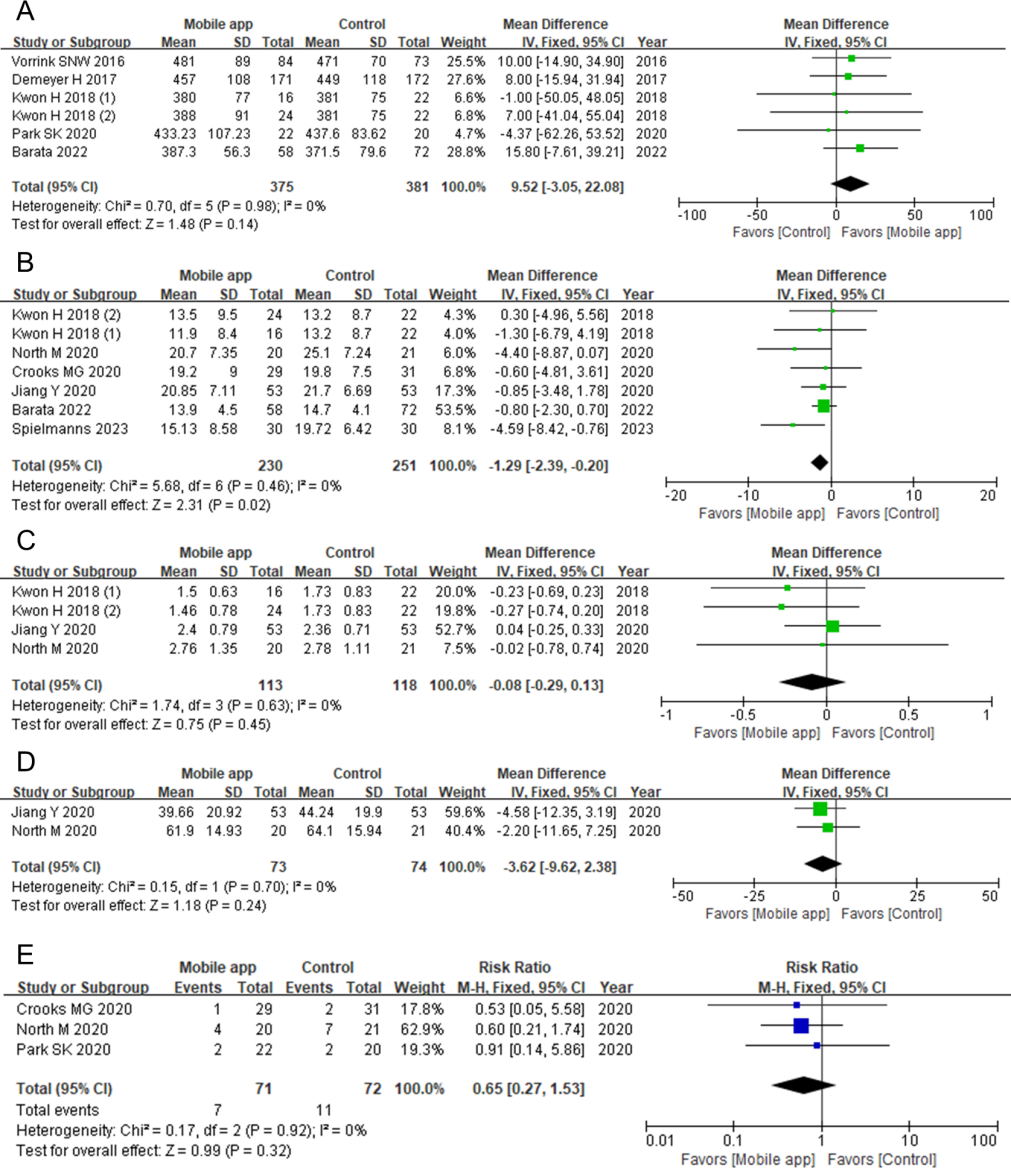
Forest plots of study outcomes for the intervention and control groups [23-31]. A: 6-minute walk test distance. B: COPD (chronic obstructive pulmonary disease) Assessment Test score. C: Modified Medical Research Council dyspnea scale score. D: St. George Respiratory Questionnaire. E: Hospitalization resulting from disease exacerbation. “Kwon H 2018 (1)” denotes the fixed regimen group, and “Kwon H 2018 (2)” denotes the fixed-interactive regimen group. IV: inverse variance; M-H: Mantel-Haenszel.

Figure 3 shows the meta-analysis of study outcomes. In terms of statistical heterogeneity, the chi-square test and *I*^2^ statistic for each meta-analysis showed no important heterogeneity. Exercise capacity was reported in various forms, including 6MWDs, ISWT results, the number of steps per day, and metabolic equivalents, in 8 studies [23-25,27,29-32]. Wang et al [32] reported on the ISWT only, and Crooks et al [24] and Spielmanns et al [30] reported the number of steps per day only. Thus, the 6MWD, which was used in 5 studies, was included in the meta-analysis [23,25,27,29,31]; there was no statistically significant difference between groups (mean difference 9.52, 95% CI −3.05 to 22.08 m; *P*=.14).

CAT scores were reported in 7 studies [23-28,30]; however, Demeyer et al [25] reported the CAT scores as medians and IQRs. Thus, the CAT scores from 6 studies were analyzed [23,24,26-28,30]. The CAT scores of the intervention group were significantly lower than those of the control group (mean difference –1.29, 95% CI −2.39 to −0.20; *P*=.02). Dyspnea was measured by using the mMRC dyspnea scale in 3 studies [26-28], and the scores did not significantly differ between groups (mean difference −0.08, 95% CI −0.29 to 0.13; *P*=.45). The quality of life was assessed in 6 studies, using various questionnaires [24-26,28,29,31], and SGRQ scores were reported in 2 trials [26,28]; there was no statistical difference in these scores between groups (mean difference −3.62, 95% CI −9.62 to 2.38; *P*=.24).

The exacerbation of COPD was reported as outpatient clinic visits, emergency room visits, or hospitalizations in 4 studies [24,25,28,29]. Among them, hospitalizations were reported in 3 studies [24,28,29]. The frequency of hospitalization was not statistically different between groups (risk ratio 0.65, 95% CI 0.27 to 1.53; *P*=.32).

We also performed a subgroup analysis for the 6MWDs and CAT scores based on the baseline study results (6MWDs: ≥400 m vs <400 m; CAT scores: ≥20 vs <20) [23-31]. The subgroup analysis did not show statistically significant differences (all *P* values were >.05). Furthermore, we performed a subgroup analysis for the CAT scores based on the rehabilitation programs (exercise program only vs exercise and self-management programs) [23,24,26-28,30]. Among studies offering both exercise and self-management programs, the CAT scores of the intervention group were significantly lower than those of the control group (mean difference −2.16, 95% CI −3.93 to −0.39; *P*=.02; Figure S2 in Multimedia Appendix 3 [23-31]).

## Discussion

### Principal Results and Implications

We reviewed and described the clinical outcomes of mobile app–based pulmonary rehabilitation in patients with COPD. Participants and interventions were heterogeneous in their characteristics; however, participants who underwent mobile app–based pulmonary rehabilitation showed favorable exercise capacity, symptom score, quality of life, and hospitalization outcomes when compared to participants who underwent conventional pulmonary rehabilitation. In the meta-analysis, the 6MWDs, mMRC dyspnea scale scores, SGRQ scores, and exacerbations in the mobile app–based pulmonary rehabilitation group were not inferior to those in the control group, and the CAT scores were superior to those in the control group. Considering the difficulties in practicing conventional center-based pulmonary rehabilitation, mobile app–based pulmonary rehabilitation may be a useful treatment option when conventional pulmonary rehabilitation is not feasible.

### Mobile App–Based Pulmonary Rehabilitation

Pulmonary rehabilitation has been traditionally delivered in outpatient, inpatient, and community settings, comprising ≥2 sessions per week for at least 4 weeks [14]. In 2015, the American Thoracic Society/European Respiratory Society policy statement requested researchers to adopt alternative formats for pulmonary rehabilitation, demonstrate clinical outcomes that are at least comparable to those of traditional pulmonary rehabilitation programs, and evaluate cost-effectiveness and safety [36]. Since then, clinical trials have reported data on the clinical outcomes and safety of pulmonary rehabilitation program models, including home-based rehabilitation, telerehabilitation, web-based rehabilitation, community rehabilitation, primary care rehabilitation, rehabilitation requiring minimal resources, and combined heart failure/pulmonary rehabilitation models [22]. Mobile app–based pulmonary rehabilitation can be regarded as a type of telehealth intervention [20] that provides health care at a distance through telecommunications or web-based technologies [37]. It may improve the accessibility of pulmonary rehabilitation for patients with chronic respiratory diseases by providing health care access and services for patients who are geographically or socially isolated, are engaged with full‐time work, or are hard to transport due to the disease or comorbidities [20].

### Further Development of Apps

Various types of apps were used in the studies. Some authors used newly developed apps, and others used myCOPD or the social messenger app WeChat [24,26,28]. Some apps, such as myCOPD, provided self-management programs for COPD, including education and symptom management programs [24,28]; however, other apps provided only exercise programs [25,27]. Although this study focused on clinical improvements in participants who underwent pulmonary rehabilitation, it should also be considered that overall self-management programs, such as disease education and symptom management programs, have affected clinical outcomes. However, *pulmonary rehabilitation* is defined as a comprehensive intervention that includes exercise training, education, and behavior change [12]. Recently, Holland et al [22] suggested that desirable components of pulmonary rehabilitation should include education, self-management training, smoking cessation, and an action plan for exacerbation, as well as a home exercise program. Therefore, apps that provide both exercise programs and self-management programs should be included in mobile app–based pulmonary rehabilitation.

Considering the challenges in center-based pulmonary rehabilitation and the shortage of health care resources, home-based pulmonary rehabilitation has been studied as an alternative to center-based pulmonary rehabilitation [38-43]. However, compliance to pulmonary rehabilitation is an important issue in home-based pulmonary rehabilitation, and a lack of motivation is an important reason for poor compliance [44]. In a study of home-based pulmonary rehabilitation without supervision, patients with good compliance showed significant improvements in CAT scores, BODE index scores, and FEV_1_ values when compared to patients with poor compliance [45]. Similarly, Crooks et al [24] described that there was an estimated −0.22 (95% CI −0.74 to −0.31) decrease in the CAT score for every 7-day increase in app use (adjusted for baseline CAT score, COPD severity, and study site). However, North et al [28] reported that as time passed, the number of app users decreased in mobile app–based pulmonary rehabilitation. Therefore, patients are required to steadily run the app and perform pulmonary rehabilitation to achieve clinical improvement. Various methods were used in studies to enhance compliance, such as sending text messages with activity proposals to participants, contacting participants via telephone, providing incentives, and having participants communicate with other participants [25,26,29,31,32]. Additionally, activity level (step counts) was monitored by using a pedometer, and feedback was provided to participants [25,29,31,32]. In real-world practice, health care interventions and action plans should be considered in cases of poor compliance because poor compliance might reflect deconditioning or acute exacerbation among patients [22,26,44].

### Further Development of Rehabilitation Programs

In clinical practice, exercise levels in pulmonary rehabilitation should be individualized according to patients’ exercise capacity [12,13]. Therefore, in mobile app–based pulmonary rehabilitation, maintaining appropriate exercise levels is a matter of concern. Some apps provided adjustable exercise regimens according to the changes in participants’ exercise capacity [25,27,31,32]. Kwon et al [27] designed exercise regimens in which the exercise levels were adjusted according to the maximum walking speed in the 6-minute walk test and the degree of breathing difficulty after exercise. Vorrink et al [31] designed physical activity goals that were set according to average steps per day. To maintain appropriate exercise levels, apps should provide adjustable and individualized exercise programs based on patients’ exercise capacity and activity level data that are collected via wearable devices or smartphone-mounted sensors.

Considering the study designs included in this review, it is important to develop strategies for improving compliance to rehabilitation and design individualized exercise programs to achieve significant improvements in clinical outcomes in future studies. Moreover, most studies (6/10, 60%) had rather small sample sizes (<100 individuals) for demonstrating the efficacy of pulmonary rehabilitation programs [24,27-30,32]. In addition, most studies (8/10, 80%) did not provide data regarding app usage, which could have been used in the subgroup analysis related to compliance [23,25-27,29-32]. Therefore, further studies with larger sample sizes and data on app usage are needed.

Nutrition support is also an important part of pulmonary rehabilitation [13,22]. In this review, some of the included apps provided disease education; however, a nutrition support program was not provided [24,26,28-30,35]. Nutrition support may help patients with COPD to maintain an adequate BMI and increase their muscle mass [13,22]. Exercise training that is accompanied by nutrition support might improve respiratory sarcopenia and enhance clinical benefits [15]; thus, further studies are needed in this area.

### Clinical Outcomes and Prognosis

Exercise capacity and physical activity data can be used to predict the prognosis of patients with COPD. Exercise capacity inversely correlates with mortality in patients with COPD [46]. Physical activity also inversely correlates with exacerbation and mortality in patients with COPD [47]. Some of the reviewed studies reported physical activity as daily step counts, and these data were too widely distributed to be synthesized in the meta-analysis [24,30]. Moreover, the 6MWDs were not significantly different in the meta-analysis (*P*=.14), and Wang et al [32] reported improvements in the ISWT and limb muscle mass in the intervention group. Thus, further studies are required to ascertain whether mobile app–based pulmonary rehabilitation can improve exercise capacity and physical activity in patients with COPD.

In some studies, we noticed that mobile app–based pulmonary rehabilitation improved quality of life, including the SGRQ, Clinical COPD Questionnaire, and Chronic Respiratory Disease Questionnaire scores [24-26,28,29,31]. Among these, the CAT scores significantly improved in the intervention group, as per the meta-analysis (*P*=.02) [23,24,26-28,30]. The CAT scores correlated with the severity of airflow limitation and disease exacerbation in patients with COPD [48,49]. Taken together, mobile app–based pulmonary rehabilitation programs might improve clinical outcomes, such as acute exacerbation and mortality. Unfortunately, in the meta-analysis, there was no statistically significant difference in acute exacerbations between groups (*P*=.32) because the study periods (range 3-6 mo) might have been too short to observe acute exacerbations [24,28,29]. Therefore, further studies with long-term follow-ups are required to evaluate the effect of mobile app–based pulmonary rehabilitation on acute exacerbations and mortality.

### Limitations

First, discrepancies in the baseline status of participants were one of the main obstacles in synthesizing clinical outcomes. In the study by North et al [28], participants were evaluated after hospitalization with an acute exacerbation. In the studies by Vorrink et al [31] and Wang et al [32], physical activity in participants with COPD was evaluated after pulmonary rehabilitation. Despite this heterogeneity, participants who underwent mobile app–based pulmonary rehabilitation showed consistently favorable results for clinical parameters. Second, discrepancies in the clinical parameters were also an obstacle in synthesizing clinical outcomes. Among the various parameters for exercise capacity, a meta-analysis could be performed on the 6MWD, as it was used in half of the reviewed studies (5/10, 50%) [23,25,27,29,31], and the 6MWD is a well-established surrogate marker in patients with COPD [1,50]. Questionnaires about quality of life, including the SGRQ, EQ-5D-5L, Clinical COPD Questionnaire, and Chronic Respiratory Disease Questionnaire, also showed generally favorable results in patients who underwent mobile app–based pulmonary rehabilitation [24-26,28,29,31]. Although clinical outcomes did not reflect statistically significant improvement in participants who underwent mobile app–based pulmonary rehabilitation and decisive evidence was hard to derive, this study showed that clinical outcomes generally favored mobile app–based pulmonary rehabilitation. Considering the difficulties with center-based pulmonary rehabilitation in real-world practice, mobile app–based pulmonary rehabilitation could be a reasonable alternative to conventional pulmonary rehabilitation.

### Conclusion

In conclusion, this review shows that many mobile apps have been applied to pulmonary rehabilitation for patients with COPD. There were discrepancies in the baseline participant characteristics and interventions among studies. Nevertheless, in some studies, patients who participated in mobile app–based pulmonary rehabilitation showed favorable exercise capacity, symptom score, quality of life, and hospitalization outcomes when compared with those who underwent conventional pulmonary rehabilitation. In the meta-analysis, the 6MWDs, mMRC dyspnea scale scores, SGRQ scores, and exacerbations in the mobile app–based pulmonary rehabilitation group were not inferior to those in the control group, and the CAT scores were superior to those in the control group. Therefore, in real-world practice, mobile app–based pulmonary rehabilitation can be a useful treatment option when conventional center-based pulmonary rehabilitation is not feasible.

## Supplementary material

10.2196/41753 Multimedia Appendix 1 PICOTS-SD (population, intervention, comparison, outcomes, time, setting, and study design) search strategy for mobile apps for patients with chronic pulmonary disease.

10.2196/41753 Multimedia Appendix 2 Supplementary tables for the inclusion criteria, exclusion criteria, and clinical outcomes of the included studies.

10.2196/41753 Multimedia Appendix 3 Supplementary figures for the outcomes of included studies.

10.2196/41753 Checklist 1 PRISMA (Preferred Reporting Items for Systematic Reviews and Meta-Analyses) checklist.

## Abbreviations

6MWD: 6-minute walk test distance
BODE: BMI, Airflow Obstruction, Dyspnea, and Exercise Capacity
CAT: COPD Assessment Test
COPD: chronic obstructive pulmonary disease
FEV_1_: forced expiratory volume in 1 second
GOLD: Global Initiative for Chronic Obstructive Lung Disease
ISWT: incremental shuttle walk test
mMRC: modified Medical Research Council
PICOTS-SD: population, intervention, comparison, outcomes, time, setting, and study design
SGRQ: St. George Respiratory Questionnaire

## References

[1] Vogelmeier CF, Criner GJ, Martinez FJ (2017). Global strategy for the diagnosis, management, and prevention of chronic obstructive lung disease 2017 report. GOLD executive summary. Am J Respir Crit Care Med.

[2] GBD 2015 Chronic Respiratory Disease Collaborators (2017). Global, regional, and national deaths, prevalence, disability-adjusted life years, and years lived with disability for chronic obstructive pulmonary disease and asthma, 1990-2015: a systematic analysis for the Global Burden of Disease Study 2015. Lancet Respir Med.

[3] López-Campos JL, Tan W, Soriano JB (2016). Global burden of COPD. Respirology.

[4] Lee EG, Rhee CK (2021). Epidemiology, burden, and policy of chronic obstructive pulmonary disease in South Korea: a narrative review. J Thorac Dis.

[5] Yoon J, Seo H, Oh IH, Yoon SJ (2016). The non-communicable disease burden in Korea: findings from the 2012 Korean Burden of Disease Study. J Korean Med Sci.

[6] Gross NJ (2001). Extrapulmonary effects of chronic obstructive pulmonary disease. Curr Opin Pulm Med.

[7] Almagro P, Calbo E, de Echagüen AO (2002). Mortality after hospitalization for COPD. Chest.

[8] Yohannes AM, Baldwin RC, Connolly M (2002). Mortality predictors in disabling chronic obstructive pulmonary disease in old age. Age Ageing.

[9] Waschki B, Kirsten A, Holz O (2011). Physical activity is the strongest predictor of all-cause mortality in patients with COPD: a prospective cohort study. Chest.

[10] Celli BR, Cote CG, Marin JM (2004). The body-mass index, airflow obstruction, dyspnea, and exercise capacity index in chronic obstructive pulmonary disease. N Engl J Med.

[11] Martinez FJ, Han MK, Andrei AC (2008). Longitudinal change in the BODE index predicts mortality in severe emphysema. Am J Respir Crit Care Med.

[12] Spruit MA, Singh SJ, Garvey C (2013). An official American Thoracic Society/European Respiratory Society statement: key concepts and advances in pulmonary rehabilitation. Am J Respir Crit Care Med.

[13] Bolton CE, Bevan-Smith EF, Blakey JD (2013). British Thoracic Society guideline on pulmonary rehabilitation in adults. Thorax.

[14] McCarthy B, Casey D, Devane D, Murphy K, Murphy E, Lacasse Y (2015). Pulmonary rehabilitation for chronic obstructive pulmonary disease. Cochrane Database Syst Rev.

[15] Nagano A, Wakabayashi H, Maeda K (2021). Respiratory sarcopenia and sarcopenic respiratory disability: concepts, diagnosis, and treatment. J Nutr Health Aging.

[16] Casaburi R, Patessio A, Ioli F, Zanaboni S, Donner CF, Wasserman K (1991). Reductions in exercise lactic acidosis and ventilation as a result of exercise training in patients with obstructive lung disease. Am Rev Respir Dis.

[17] Maltais F, LeBlanc P, Simard C (1996). Skeletal muscle adaptation to endurance training in patients with chronic obstructive pulmonary disease. Am J Respir Crit Care Med.

[18] Johnston KN, Young M, Grimmer KA, Antic R, Frith PA (2013). Barriers to, and facilitators for, referral to pulmonary rehabilitation in COPD patients from the perspective of Australian general practitioners: a qualitative study. Prim Care Respir J.

[19] Augustine A, Bhat A, Vaishali K, Magazine R (2021). Barriers to pulmonary rehabilitation - a narrative review and perspectives from a few stakeholders. Lung India.

[20] Cox NS, Dal Corso S, Hansen H (2021). Telerehabilitation for chronic respiratory disease. Cochrane Database Syst Rev.

[21] Tsutsui M, Gerayeli F, Sin DD (2021). Pulmonary rehabilitation in a post-COVID-19 world: telerehabilitation as a new standard in patients with COPD. Int J Chron Obstruct Pulmon Dis.

[22] Holland AE, Cox NS, Houchen-Wolloff L (2021). Defining modern pulmonary rehabilitation. Ann Am Thorac Soc.

[23] Barata PI, Crisan AF, Maritescu A (2022). Evaluating virtual and inpatient pulmonary rehabilitation programs for patients with COPD. J Pers Med.

[24] Crooks MG, Elkes J, Storrar W (2020). Evidence generation for the clinical impact of myCOPD in patients with mild, moderate and newly diagnosed COPD: a randomised controlled trial. ERJ Open Res.

[25] Demeyer H, Louvaris Z, Frei A (2017). Physical activity is increased by a 12-week semiautomated telecoaching programme in patients with COPD: a multicentre randomised controlled trial. Thorax.

[26] Jiang Y, Liu F, Guo J (2020). Evaluating an intervention program using WeChat for patients with chronic obstructive pulmonary disease: randomized controlled trial. J Med Internet Res.

[27] Kwon H, Lee S, Jung EJ (2018). An mHealth management platform for patients with chronic obstructive pulmonary disease (efil breath): randomized controlled trial. JMIR Mhealth Uhealth.

[28] North M, Bourne S, Green B (2020). A randomised controlled feasibility trial of e-health application supported care vs usual care after exacerbation of COPD: the RESCUE trial. NPJ Digit Med.

[29] Park SK, Bang CH, Lee SH (2020). Evaluating the effect of a smartphone app-based self-management program for people with COPD: a randomized controlled trial. Appl Nurs Res.

[30] Spielmanns M, Gloeckl R, Jarosch I (2023). Using a smartphone application maintains physical activity following pulmonary rehabilitation in patients with COPD: a randomised controlled trial. Thorax.

[31] Vorrink SNW, Kort HSM, Troosters T, Zanen P, Lammers JWJ (2016). Efficacy of an mHealth intervention to stimulate physical activity in COPD patients after pulmonary rehabilitation. Eur Respir J.

[32] Wang CH, Chou PC, Joa WC (2014). Mobile-phone-based home exercise training program decreases systemic inflammation in COPD: a pilot study. BMC Pulm Med.

[33] Bolton CE, Waters CS, Peirce S, Elwyn G, EPSRC and MRC Grand Challenge Team (2011). Insufficient evidence of benefit: a systematic review of home telemonitoring for COPD. J Eval Clin Pract.

[34] Sobnath DD, Philip N, Kayyali R (2017). Features of a mobile support app for patients with chronic obstructive pulmonary disease: literature review and current applications. JMIR Mhealth Uhealth.

[35] Spielmanns M, Boeselt T, Huber S (2020). Impact of a smartphone application (KAIA COPD app) in combination with activity monitoring as a maintenance program following pulmonary rehabilitation in COPD: the protocol for the AMOPUR Study, an international, multicenter, parallel group, randomized, controlled study. Trials.

[36] Rochester CL, Vogiatzis I, Holland AE (2015). An official American Thoracic Society/European Respiratory Society policy statement: enhancing implementation, use, and delivery of pulmonary rehabilitation. Am J Respir Crit Care Med.

[37] Global Observatory for eHealth (2016). Global diffusion of eHealth: making universal health coverage achievable: report of the third global survey on eHealth. World Health Organization.

[38] Na JO, Kim DS, Yoon SH (2005). A simple and easy home-based pulmonary rehabilitation programme for patients with chronic lung diseases. Monaldi Arch Chest Dis.

[39] Lee S, Kim C, Jin YS (2013). Effects of home-based pulmonary rehabilitation with a metronome-guided walking pace in chronic obstructive pulmonary disease. J Korean Med Sci.

[40] Coultas DB, Jackson BE, Russo R (2018). Home-based physical activity coaching, physical activity, and health care utilization in chronic obstructive pulmonary disease: chronic obstructive pulmonary disease self-management activation research trial secondary outcomes. Ann Am Thorac Soc.

[41] Holland AE, Mahal A, Hill CJ (2017). Home-based rehabilitation for COPD using minimal resources: a randomised, controlled equivalence trial. Thorax.

[42] Chaplin E, Hewitt S, Apps L (2017). Interactive web-based pulmonary rehabilitation programme: a randomised controlled feasibility trial. BMJ Open.

[43] Horton EJ, Mitchell KE, Johnson-Warrington V (2018). Comparison of a structured home-based rehabilitation programme with conventional supervised pulmonary rehabilitation: a randomised non-inferiority trial. Thorax.

[44] Li Y, Qian H, Yu K, Huang Y (2020). Nonadherence in home-based pulmonary rehabilitation program for COPD patients. Can Respir J.

[45] Lee JH, Lee HY, Jang Y (2020). Efficacy of unsupervised home-based pulmonary rehabilitation for patients with chronic obstructive pulmonary disease. Int J Chron Obstruct Pulmon Dis.

[46] Oga T, Nishimura K, Tsukino M, Sato S, Hajiro T (2003). Analysis of the factors related to mortality in chronic obstructive pulmonary disease: role of exercise capacity and health status. Am J Respir Crit Care Med.

[47] Garcia-Rio F, Rojo B, Casitas R (2012). Prognostic value of the objective measurement of daily physical activity in patients with COPD. Chest.

[48] Lee SD, Huang MS, Kang J (2014). The COPD Assessment Test (CAT) assists prediction of COPD exacerbations in high-risk patients. Respir Med.

[49] Ghobadi H, Ahari SS, Kameli A, Lari SM (2012). The relationship between COPD Assessment Test (CAT) scores and severity of airflow obstruction in stable COPD patients. Tanaffos.

[50] Holland AE, Spruit MA, Troosters T (2014). An official European Respiratory Society/American Thoracic Society technical standard: field walking tests in chronic respiratory disease. Eur Respir J.

