# Supplementary material for: Clinical Efficacy of Mobile App–Based, Self-Directed Pulmonary Rehabilitation for Patients With Chronic Obstructive Pulmonary Disease: Systematic Review and Meta-Analysis
Source: JMIR Mhealth Uhealth. 2024 Jan 4;12:e41753. doi: 10.2196/41753 (PMC10786334; doi:10.2196/41753)
Supplement: Multimedia Appendix 1 [file mhealth-v12-e41753-s001.docx]

1. **PICOTS-SD (population, intervention, comparison, outcomes, time, setting, and study design) search strategy for mobile apps for patients with chronic pulmonary disease.**
2. **Population**

- Adult patients with chronic pulmonary (lung, respiratory) disease
- Chronic pulmonary (lung, respiratory) diseases:
  1) COPD, chronic obstructive pulmonary disease, emphysema, chronic bronchitis
  2) Asthma, bronchial asthma
  3) Bronchiectasis, cystic fibrosis
  4) ILD, interstitial lung disease, IIP, idiopathic interstitial pneumonia, IPF, idiopathic pulmonary fibrosis, NSIP, nonspecific interstitial pneumonia, RB-ILD, Respiratory bronchiolitis interstitial lung disease, DIP, Desquamative interstitial pneumonia, OP, organizing pneumonia, AIP, acute interstitial pneumonia, CTD-ILD, connective tissue disease-related interstitial lung disease, pneumoconiosis, hypersensitivity pneumonitis
  5) Critical care, intensive care, critical illness, critically ill, ICU

1. **Intervention**

- Mobile application, mobile apps, smartphone application, smartphone apps, mobile pulmonary rehabilitation

1. **Comparison**

- No treatment, placebo, basic supportive care, standard (conventional) medical treatment, education

1. **Outcomes**

- Body weight, BMI (body mass index)
- 6-minute walk (walking) test: distance, saturation
- Endurance shuttle walk (walking) test, incremental shuttle walk (walking) test
- CPET (cardiopulmonary exercise test)
- Pulmonary function test: FEV_1_, FVC, DLCO
- Acute exacerbation, hospitalization, mortality
- HRQOL (health-related quality of life), QOL (quality of life)
  COPD: CAT, SGRQ, SGRQ-C, EQ-5D, CCQ, CRQ
  Asthma: ACT, AQLQ, SGRQ, EQ-5D
  Bronchiectasis: SGRQ, LCQ, CRQ
  ILD: SGRQ, SGRQ-I, K-BILD, SF-36, SOBQ
  Critical care: SGRQ, CRQ, SF-36

1. **Time**

- 2007–2021

1. **Setting**

- No limitations

1. **Study design**

- Randomized controlled trial, quasi-randomized trial, non-randomized trial/quasi-experimental study, controlled before-and-after study, before-and-after study
