# Supplementary material for: Clinical Efficacy of Mobile App–Based, Self-Directed Pulmonary Rehabilitation for Patients With Chronic Obstructive Pulmonary Disease: Systematic Review and Meta-Analysis
Source: JMIR Mhealth Uhealth. 2024 Jan 4;12:e41753. doi: 10.2196/41753 (PMC10786334; doi:10.2196/41753)
Supplement: Multimedia Appendix 2 [file mhealth-v12-e41753-s002.docx]

**Table S1.** Inclusion and exclusion criteria of the included studies.

| First author | Inclusion criteria | Exclusion criteria |
| --- | --- | --- |
| Barata PI [23] | Age > 45 years, will participate, no exacerbation in the last three months,  no prior rehabilitation in the last three months, former smoking history, non-smoking  status, owning a mobile smartphone, able to use a smartphone, stationary bicycle at home (for the online group), owning a pulse oximeter. | Exacerbation in the last three months, other comorbidities that could interfere with their current health status, use of medication that could affect exercise response, active smoking status, musculoskeletal conditions that could impair exercise, an impaired vision that could affect the use of the mobile application, not having a stationary bicycle at home, a cognitive impairment that could affect the understanding of the exercises. |
| Crooks MG [24] | Aged 40–80 years with either mild–moderate COPD (forced expiratory volume in 1 s (FEV_1_) >50% predicted and FEV_1_/forced vital capacity ratio <70%) or COPD of any severity diagnosed within the past 12 months  Current or ex-smokers with internet access and able to use a web platform in English | A COPD exacerbation within 4 weeks before enrolment  housebound  Another medical condition considered by the investigator to confound study outcomes |
| Demeyer H [25] | Physician-based diagnosis of COPD  Age >40 with a smoking history of at least 10 pack-years  Not actively participating in a pulmonary rehabilitation program at the moment of inclusion (or did not plan to start)  Stable patients as well as patients with an acute exacerbation in the last month  Patients using walking aids or those on long-term oxygen treatment | Any comorbidity limiting a normal activity patterns  Another respiratory disease as a primary diagnosis  Unable to understand or operate a smartphone device |
| Jiang Y [26] | Aged 60 years and older  Confirmed diagnosis of COPD according to the diagnosis and treatment guidelines for chronic obstructive pulmonary disease, forced expiratory volume in 1 second (FEV_1_)/forced vital capacity (FVC) ratio of <0.7, FEV_1_<80% predicted  Use of WeChat for effective communication | Patients with mental disorders, cognitive disorders, and limb dysfunction; with unstable heart disease or arrhythmia requiring drug intervention; with a history of myocardial infarction or cerebral infarction in the previous year; too weak to perform the muscle strength test; with hypertension that could not be controlled with drugs; with a history of syncope after exercise |
| Kwon H [27] | Patients with COPD  (1) Age>20 years  (2) Postbronchodilator forced expiratory volume in 1 second (FEV_1_) of <80% compared with the reference range  (3) Ability to walk >150 m in a 6MWT  (4) Android smartphone owner | Patients who were unable to follow the exercise regimen |
| North M [28] | A primary COPD diagnosis as defined by the NICE guidelines and using an inhaled device  Age 45 years or older  Current or ex-smoker for over 10 years  Ability to access and use an internet enabled device. | An allergy to saccharin due to it being contained within the placebo inhalers |
| Park SK [29] | (a) COPD  (b) Aged 45 years or old  (c) Classified as either GOLD Stage 1, 2, or 3  (d) Smartphone and could text messages  (e) Ability to communicate. | (a) Psychiatric disorder  (b) Hospitalization and discharge within 8 weeks due to a COPD exacerbation  (c) Oxygen saturation<93% in a stable state  (d) Saturation levels that decreased to 85% after a six-minute walk test (6MWT)  (e) Severe respiratory symptoms in a stable state  (f) Pulmonary rehabilitation within 12 months  (g) Other diseases that made physical activity and/or exercise difficult  (h) Usage of assistive devices to walk or problems with balance. |
| Spielmanns M [30] | COPD patients willing and able to sign the informed consent form for use of their pseudonymized clinical data within the scope of the present interventional trial  COPD patients who have completed an in-hospital pulmonary rehabilitation program for an average duration of 3 weeks  Diagnosis of COPD, defined as forced expiratory volume in 1 s/forced vital capacity (FEV1/FVC) < 70% predicted, FEV1< 80% predicted after bronchodilation, with or without chronic symptoms (cough, sputum production) corresponding to GOLD stage II–IV  Completion of an inpatient pulmonary rehabilitation program  Completion of the screening period and fulfillment of the randomization criteria as defined by the protocol  Ability to use a smartphone and smartphone apps  Willingness to wear an activity tracker during the 6-month study period  Age ≥ 40 years of age  Knowledge of German language to understand the study material, assessments, and contents of the COPD app | The patient is unable to conduct the exercise training program due to physical, cognitive, or safety reasons, as judged by the investigators, e.g., lower limb joint surgery within the preceding 3 months, unstable cardiac diseases, predominant neurological limitations, and planned surgical or other interventions disturbing the study intervention  Significant psychiatric disorders, legal incapacity, or limited legal capacity. Patient participation in another clinical trial with an investigational medication within 30 days prior to study entry  Patients already using the KAIA COPD app |
| Vorrink SNW [31] | Patients diagnosed with COPD  Global Initiative for Chronic Obstructive Lung Disease (GOLD) stage 2 or 3 (forced expiratory volume in 1 s (FEV1) 30–<80%, FEV_1_/forced vital capacity (FVC) <70% after bronchodilatation)  Age≥40 years  Completion of a PR program of 3 months within the past 6 months  Living independently | Comorbidity that greatly influences physical activity  Usage of an assistive device for physical activity (e.g., walker or mobility scooter)  Intermittent cessation of the PR program  Exacerbation resulting in a hospital admission in the 6 months prior to the commencement of the study. |
| Wang CH [32] | Diagnosis of COPD [with a ratio of forced expiratory volume in one second (FEV_1_) to forced vital capacity (FVC) less than 0.7 after bronchodilators]  The grading of moderate-to-severe airflow limitation according to GOLD criteria  Stable within three months prior to enrollment. | Requirement for oxygen therapy  Presence of symptomatic cardiovascular diseases or severe systemic diseases or musculoskeletal conditions with exercise performance limitation |

**Table S2.** Clinical outcomes of the included studies.

1. Barata PI [23]

| Clinical outcomes | Intervention arm | Control arm |
| --- | --- | --- |
| Primary outcome |  |  |
| FVC (%) | 71.0 ± 6.8 to 71.4 ± 6.6 | 70.8 ± 5.9 to 70.1 ± 5.9 |
| FEV1 (%) | 41.7 ± 4.6 to 42.2 ± 4.6 | 42.5 ± 4.6 to 43.1 ± 4.5 |
| FEV1/FVC (%) | 44.2 ± 6.5 to 44.5 ± 6.2 | 44.9 ± 5.7 to 45.2 ± 5.7 |
| MIP (cmH_2_O) | 55.7 ± 12.1 to 59.9 ± 12.3 | 55.7 ± 15.8 to 62.5 ± 16.6 |
| MEP (cmH_2_O) | 80.2 ± 13.6 to 83.3 ± 13.1 | 82.2 ± 12.3 to 86.8 ± 12.5 |
| 6MWT (m) | 342.9 ± 61.9 to 387.3 ± 56.3 | 340.5 ± 85.0 to 371.5 ± 79.6 |
| CAT | 20.1 ± 5.3 to 13.9 ± 4.5 | 19.5 ± 5.1 to 14.7 ± 4.1 |
| mMRC (mean rank) | 39.4 to 19.5 | 45.25 to 27.75 |

Data are presented as the mean ± standard deviation or number (%), unless otherwise indicated.

FVC: forced vital capacity, FEV1: forced expiratory volume in the first second, MIP: maximal inspiratory pressure, MEP: maximal expiratory pressure, 6MWT: 6-minute walking test, CAT: COPD assessment test, mMRC: modified Medical Research Council scale.

1. Crooks MG [24]

| Clinical outcomes | Intervention arm | Control arm |
| --- | --- | --- |
| Primary outcome |  |  |
| CAT | 21.5 ± 8.0 to 19.2 ± 9.0 | 19.8 ± 5.4 to 19.8 ± 7.5 |
| ≥1 critical error inhaler error | 21 (72.4)  Difference at 3 months compared with baseline: −0.3 (0.70) | 18 (58.1)  Difference at 3 months compared with baseline: 0.1 (0.71) |
| Average inhaler errors | 1.1 ± 1.3  Difference at 3 months compared with baseline: −0.3 (1.61) | 1.0 ± 1.1  Difference at 3 months compared with baseline: −0.1 (1.20) |
| Secondary outcome |  |  |
| PAM score | 59.9 ± 15.9  Difference at 3 months compared with baseline: −0.7 (14.28) | 69.0 ± 13.8  Difference at 3 months compared with baseline: −3.5 (13.07) |
| SEAMS | 32.8 ± 5.7  Difference at 3 months compared with baseline: 1.0 (0.00) | 33.8 ± 4.9  Difference at 3 months compared with baseline: 0.0 (−3.00) |
| EQ5D 5L | 0.6 ± 0.3  Difference at 3 months compared with baseline: 0.1 (0.23) | 0.7 ± 0.2  Difference at 3 months compared with baseline: 0.0 (0.18) |
| Other outcomes |  |  |
| Exacerbations | 18 exacerbations, 2 ER visits, 1 hospitalization | 11 exacerbations, 1 ER visit, 2 hospitalizations |
| Number of steps per day | 4948.7 ± 1667.6 (n=5) to 5458.3 ± 2266.4 (n=4) | 9060 ± 5135.1 (n=9) to 10,762 ± 7199.2 (n=9) |
| Adverse events | 5 | 7 |

Data are presented as the mean ± standard deviation or number (%), unless otherwise indicated.

CAT: COPD assessment test, EQ5D 5L: EuroQol 5 dimensions 5-level questionnaire, VAS: visual analog scale, PAM: patient activation measurement, SEAMS: Self-Efficacy for Appropriate Medication Use Scale.

1. Demeyer H [25]

| Clinical outcomes | Intervention arm | Control arm |
| --- | --- | --- |
| Primary outcome |  |  |
| Number of steps per day | 4305 [2841–5851] to 4767 [3080–7949] | 4643 [2932–6955] to 4059 [2624–6332] |
| Secondary outcome |  |  |
| Time in at least moderately intense physical activity (min) | 14 [5–26] to 18 [6–48] | 15 [5–35] to 14 [3–32] |
| Walking time (min) | 69 ± 34  Difference at 3 months compared with baseline: 7 (95% CI 8 to 12) | 72 ± 36  Difference at 3 months compared with baseline: -10 (95% CI -14 to -6) |
| Movement intensity during walking (m/s^2) | 1.82 ± 0.30  Difference at 3 months compared with baseline: 0.06 (95% CI 0.02 to 0.1) | 1.86 ± 0.36  Difference at 3 months compared with baseline: -0.03 (95% CI -0.06 to 0.01) |
| 6MWD | 444 ± 106 to 457 ± 108 | 450 ± 106 to 449 ± 118 |
| CAT | 13 [7–20] to 14 [9–19] | 13 [8–18] to 13 [9–20] |
| CCQ mental state | 1 [0–2.5] to 1 [0–2.5] | 1 [0–2] to 1 [0–2] |
| CCQ functional state | 1.5 [1–2.75] to 1.5 [1–2.75] | 1.5 [0.75–2.5] to 1.75 [0.75–2.75] |
| CCQ symptoms | 1.75 [1.25–2.5] to 1.75 [1.25–2.5] | 1.75 [1.5–2.75] to 2 [1.25–2.75] |
| At least one exacerbation | 48 (30%) | 43 (27%) |
| Lung function variables | Lung function variables during the final visit were not different from baseline variables in either group. |  |
| Musculoskeletal events | 11 | 2 |

Data are presented as the mean ± standard deviation or median [interquartile range] or number (%), unless otherwise indicated.

CI, confidence interval; 6MWD, 6-min walk distance; CAT, COPD assessment test; CCQ, Clinical COPD Questionnaire

1. Jiang Y [26]

| Clinical outcomes | Intervention arm | Control arm |
| --- | --- | --- |
| Primary outcome |  |  |
| CAT | 21.79 ± 6.85 to 20.85 ± 7.11 | 22.55 ± 6.48 to 21.70 ± 6.69 |
| Secondary outcome |  |  |
| Ex-SRES | 72.25 ± 38.38 to 80.53 ± 37.72 | 71.48 ± 40.76 to 78.25 ± 35.40 |
| mMRC | 2.79 ± 0.66 to 2.40 ± 0.79 | 2.75 ± 0.70 to 2.36 ± 0.71 |
| SGRQ-system | 53.02 ± 19.90 to 43.59 ± 23.63 | 51.12 18.63± to 45.33 ± 22.25 |
| SGRQ-activity | 56.44 ± 23.96 to 48.74 ± 24.28 | 56.87 ± 22.47 to 53.46 ± 23.06 |
| SGRQ-influence | 45.83 ± 24.27 to 33.27 ± 22.86 | 44.92 ± 18.69 to 38.63 ± 21.88 |
| SGRQ-total | 50.24 ± 20.95 to 39.66 ± 20.92 | 49.57 ± 17.52 to 44.24 ± 19.90 |

Data are presented as the mean ± standard deviation or number (%), unless otherwise indicated.

CAT: chronic obstructive pulmonary disease assessment test, Ex-SRES: Exercise Self-Regulatory Efficacy Scale, mMRC: modified Medical Research Council scale, SGRQ: St George’s Respiratory Questionnaire.

1. Kwon H [27]

| Clinical outcomes | Intervention arm | Control arm |
| --- | --- | --- |
| Primary outcome |  |  |
| 6MWT | Fixed group  369 ± 71 to 380 ± 77  Fixed-Interactive group  394 ± 88 to 388 ± 91 | 379 ± 71 to 381 ± 75 |
| mMRC | Fixed group  1.75 ± 0.68 to 1.50 ± 0.63  Fixed-Interactive group  1.50 ± 0.66 to 1.46 ± 0.78 | 1.86 ± 0.77 to 1.73 ± 0.83 |
| CAT | Fixed group  15.1 ± 7.5 to 11.9 ± 8.4 | 15.0 ± 8.7 to 13.2 ± 8.7 |
|  | Fixed-Interactive group  15.6 ± 9.1 to 13.5 ± 9.5 |  |

Data are presented as the mean ± standard deviation or number (%), unless otherwise indicated.

6MWT: 6 min walk test, mMRC: modified Medical Research Council scale, CAT: chronic obstructive pulmonary disease assessment test.

1. North M [28]

| Clinical outcomes | Intervention arm | Control arm |
| --- | --- | --- |
| Primary outcome |  |  |
| CAT | 26.0 ± 8.5 to 20.7 ± 7.35 | 28.0 ± 5.8 to 25.1 ± 7.24 |
| Secondary outcome |  |  |
| mMRC | 2.9 ± 1.3 to 2.76 ± 1.35 | 3.1 ± 1.1 to 2.78 ± 1.11 |
| PAM | 59.7 ± 11.4 to 64.7 ± 13.46 | 54.0 ± 11.2 to 56.1 ± 18.49 |
| HAD | 18.9 ± 10.6 to 15.5 ± 8.88 | 18.1 ± 6.1 to 18.1 ± 7.78 |
| SGRQ | 66.4 ± 16.6 to 61.9 ± 14.93 | 68.1 ± 13.7 to 64.1 ± 15.94 |
| WPAI questionnaire | 7.3 ± 2.0 to 6.24 ± 2.68 | 6.9 ± 2.3 to 6.50 ± 2.98 |
| VSAQ | 3.2 ± 2.7 to 2.94 ± 1.54 | 2.6 ± 1.1 to 2.95 ± 2.43 |
| Readmission rate | 0.24 ± 0.44 | 0.39 ± 0.50 |
| Number of exacerbations | 2.9 ± 1.6 to 1.06 ± 0.83 | 3.2 ± 2.0 to 1.88 ± 1.84 |
| Number of critical errors in inhaler technique | 5.1 ± 3.1 to 1.17 ± 1.70 | 5.0 ± 3.3 to 4.00 ± 4.97 |

Data are presented as the mean ± standard deviation or number (%), unless otherwise indicated.

CAT: chronic obstructive pulmonary disease assessment test, mMRC: modified Medical Research Council test for dyspnea, PAM: patient-activated measures, HAD: hospital anxiety and depression scale, SGRQ: St George’s Respiratory Questionnaire, WPAI: work productivity activity impairment, VSAQ: Veterans Specific Activity Questionnaire.

1. Park SK [29]

| Clinical outcomes | Intervention arm | Control arm |
| --- | --- | --- |
| Primary outcome |  |  |
| self-care behavior | 112.91 ± 13.34 to 122.32 ± 12.23 | 106.05 ± 14.79 to 106.70 ± 18.47 |
| Secondary outcome |  |  |
| 6MWT distance | 378.32 ± 96.96 to 433.23 ± 107.23 | 398.10 ± 78.67 to 437.60 ± 83.62 |
| Exercise (min/week) | 215.00 ± 225.51 to 267.73 ± 449.96 | 144.37 ± 129.06 to 162.50 ± 212.33 |
| Physical activity |  |  |
| Total activity count/wear time | 215.64 ± 103.16 to 275.09 ± 99.79 | 258.85 ± 105.73 to 258.59 ± 111.47 |
| Sedentary activity % time | 0.79 ± 0.10 to 0.75 ± 0.08 | 0.77 ± 0.08 to 0.77 ± 0.08 |
| LPA % time | 0.18 ± 0.09 to 0.21 ± 0.08 | 0.20 ± 0.06 to 0.19 ± 0.06 |
| MVPA % time | 0.03 ± 0.02 to 0.05 ± 0.03 | 0.04 ± 0.02 to 0.04 ± 0.03 |
| Daily step count | 5223.68 ± 2899.61 to 6546.77 ± 2354.43 | 6756.26 ± 2978.77 to 6890.39 ± 2967.73 |
| Symptom |  |  |
| Dyspnea from UCSD-SOB | 21.18 ± 16.05 to 21.45 ± 17.78 | 19.25 ± 13.83 to 19.70 ± 14.34 |
| Tension-anxiety from POMS | 4.86 ± 2.64 to 5.23 ± 3.19 | 5.75 ± 4.29 to 5.80 ± 4.61 |
| Depression from POMS | 3.55 ± 2.69 to 3.68 ± 3.29 | 5.20 ± 5.46 to 5.45 ± 6.89 |
| Health-related quality of life |  |  |
| PCS | 43.43 ± 9.00 to 43.94 ± 8.97 | 46.36 ± 5.58 to 44.95 ± 5.95 |
| MCS | 51.62 ± 8.71 to 50.10 ± 8.33 | 52.13 ± 8.49 to 49.03 ± 11.02 |
| Healthcare use due to exacerbation for 6 months |  |  |
| ED use | 1 (4.5%) | 0 (0.0%) |
| Hospitalization | 2 (9.1%) | 2 (10.0%) |
| Outpatient clinics | 3 (13.6%) | 1 (5.0%) |
| Other outcomes |  |  |
| Self-efficacy |  |  |
| SEMCD | 6.71 ± 1.93 to 6.89 ± 1.75 | 6.47 ± 1.64 to 6.69 ± 2.26 |
| Self-efficacy for managing dyspnea | 6.59 ± 2.21 to 6.73 ± 2.10 | 6.40 ± 2.10 to 6.85 ± 2.06 |
| Self-efficacy for managing exacerbation | 6.68 ± 1.94 to 6.95 ± 2.01 | 6.20 ± 2.24 to 6.75 ± 1.97 |
| Self-efficacy for maintaining exercise | 7.45 ± 1.50 to 7.77 ± 1.31 | 6.90 ± 2.05 to 6.75 ± 2.29 |
| Self-efficacy for increasing physical activity | 6.91 ± 2.14 to 7.91 ± 1.66 | 6.90 ± 1.71 to 6.75 ± 2.15 |
| Self-efficacy for decreasing sedentary time | 7.18 ± 1.76 to 7.73 ± 1.42 | 6.60 ± 2.09 to 7.05 ± 1.76 |
| Perception of control | 4.40 ± 0.96 to 4.75 ± 0.91 | 4.33 ± 1.22 to 4.68 ± 0.97 |
| Social support | 2.72 ± 0.85 to 2.73 ± 0.88 | 2.53 ± 0.92 to 2.79 ± 1.21 |

Data are presented as the mean ± standard deviation or number (%), unless otherwise indicated.

6MWT: 6 min walk test, sedentary activity % time: time spent in sedentary activity (minutes/day)/daily wear time for accelerometer, LPA: light physical activity, LPA % time: time spent in LPA (minutes/day)/daily wear time for accelerometer, MVPA: moderate to vigorous physical activity, MVPA % time: time spent in MVPA (minutes/day)/daily wear time for accelerometer, UCSD-SOB: University of California, San Diego Shortness of Breath Questionnaire, POMS: Profile of Mood States-Short Form, PCS: physical component subscale, MCS: mental component subscale, ED: emergency department, SEMCD: Self-Efficacy for Managing Chronic Diseases 6-item scale.

1. Spielmanns M [30]

| Clinical outcomes | Intervention arm | Control arm |
| --- | --- | --- |
| Primary outcome |  |  |
| Number of steps per day | 6361.4 [3401.2–8304.3] to 5016.3 [2920.3–10206.5] | 5052.21 [3531.9–8999.1] to 3105.1 [606.4–4372.0] |
| Secondary outcome |  |  |
| CAT points | 16.53 ± 7.15 to 15.13 ± 8.58 | 16.00 ± 7.12 to 19.72 ± 6.42 |
| STST repetitions | 19.07 ± 5.77 to 22.66 ± 7.23 | 16.87 ± 7.07 to 19.45 ± 9.09 |
| CRQ domains |  |  |
| Dyspnoea points | 4.54 ± 1.45 to 4.54 ± 1.65 | 4.48 ± 1.19 to 3.69 ± 1.31 |
| Fatigue points | 4.60 ± 1.22 to 4.50 ± 1.28 | 4.68 ± 1.31 to 3.72 ± 1.36 |
| Emotional function points | 5.40 ± 1.07 to 4.92 ± 1.27 | 5.14 ± 0.97 to 4.54 ± 1.40 |
| Mastery points | 5.27 ± 1.23 to 5.08 ± 1.50 | 4.97 ± 1.27 to 4.48 ± 1.51 |
| Total CRQ points | 4.95 ± 1.07 to 4.76 ± 1.30 | 4.82 ± 0.97 to 4.11 ± 1.26 |
| Feeling thermometer degrees | 61.57 ± 19 to 66.43 ± 18 | 61.57 ± 17 to 58.93 ± 21 |
| HADS-A points | 4.10 ± 3.33 to 4.43 ± 3.50 | 4.10 ± 3.50 to 5.34 ± 4.19 |
| HADS-D points | 4.23 ± 2.90 to 4.20 ± 2.95 | 4.23 ± 3.69 to 6.55 ± 5.08 |
| HADS total points | 8.33 ± 5.60 to 8.33 ± 5.60 | 8.33 ± 6.47 to 8.33 ± 6.47 |
| Duration of sleep (hours) | 7.61 ± 1.36 to 7.60 ± 1.31 | 7.73 ± 1.08 to 7.13 ± 1.69 |
| Sleep efficiency (%) | 91.71 ± 3.20 to 91.95 ± 2.28 | 90.84 ± 3.33 to 90.75 ± 2.93 |

Data are presented as the mean ± standard deviation or median [interquartile range] or number (%), unless otherwise indicated.

CAT: COPD Assessment Test, STST: Sit-to-Stand Test, CRQ: Chronic Respiratory Disease Questionnaire, HADS: Hospital Anxiety and Depression Scale, HADS-A: Hospital Anxiety and Depression Scale–Anxiety Subscale, HADS-D: Hospital Anxiety and Depression Scale–Depression Subscale,

1. Vorrink SNW [31]

| Clinical outcomes | Intervention arm | Control arm |
| --- | --- | --- |
| Primary outcome |  |  |
| Average steps per weekday | 5824 ± 3418 to 4819 ± 2883 | 5717 ± 2870 to 4950 ± 2634 |
| Average METs | 1.5 ± 0.05  Difference at 12 months compared with baseline: −0.055 (−0.15–0.04) | 1.57 ± 0.05  Difference at 12 months compared with baseline: −0.105 (−0.22–0.01) |
| Secondary outcome |  |  |
| 6MWD | 465 ± 87 to 481 ± 89 | 459 ± 73 to 471 ± 70 |
| CRQ-SAS |  |  |
| Dyspnea | 4.83 ± 1.25 to 4.63 ± 1.49 | 4.81 ± 1.3 to 4.66 ± 1.21 |
| Fatigue | 4.34 ± 1.13 to 4.14 ± 1.45 | 4.25 ± 1.15 to 4.08 ± 1.24 |
| Emotional function | 4.95 ± 1.08 to 4.94 ± 1.28 | 4.78 ± 1.24 to 4.94 ± 1.17 |
| Mastery | 5.4 ± 1.12 to 5.25 ± 1.22 | 5.32 ± 1.12 to 5.12 ± 1.23 |
| Body mass index | 27.78 ± 4.86 to 27.95 ± 4.96 | 26.77 ± 5.06 to 26.62 ± 5.07 |

Data are presented as the mean ± standard deviation or number (%), unless otherwise indicated.

MET: metabolic equivalent of task, 6MWD: 6-min walking distance, CRQ-SAS: Self-Administered Standardized Chronic Respiratory Questionnaire.

1. Wang CH [32]

| Clinical outcomes | Intervention arm | Control arm |
| --- | --- | --- |
| Primary outcome |  |  |
| ISWT | 261.5 ± 29.9 to 320.0 ± 30.7 | 251.4 ± 21.0 to 222.5 ± 28.3 |
| Limb muscle strength |  |  |
| Elbow flexion, kg | Left 11.8 ± 0.5 to 13.5 ± 0.5  Right 11.5 ± 0.6 to 14.7 ± 0.4 | Left 13.3 ± 0.7 to 13.1 ± 0.5  Right 13.6 ± 0.8 to 13.2 ± 0.6 |
| Knee extension, kg | Left 10.9 ± 0.8 to 14.7 ± 0.7  Right 10.8 ± 0.8 to 15.1 ± 0.7 | Left 12.2 ± 0.9 to 12.8 ± 0.6  Right 12.0 ± 0.8 to 12.7 ± 0.6 |
| CRP | 1531.0 ± 206.4 μg/ml to 601.1 ± 144.5 μg/ml | 1028.0 ± 213.1 μg/ml to 2080.0 ± 428.4 μg/ml |
| IL-8 | 3299.0 ± 839.4 pg/ml to 990.1 ± 175.6 pg/ml | at 6 months, plasma levels of IL-8 in the control group were higher than those the mobile group. |
| TNF-α | Did not show any difference during the period of the home exercise training program | Significantly elevated at 2, 3 and 6 months |
| IL-6 | Unchanged in the mobile phone group | 2.8 ± 0.5 pg/ml to 7.0 ± 1.0 pg/ml |

Data are presented as the mean ± standard deviation or number (%), unless otherwise indicated.
