# Supplementary material for: Clinical Efficacy of Mobile App–Based, Self-Directed Pulmonary Rehabilitation for Patients With Chronic Obstructive Pulmonary Disease: Systematic Review and Meta-Analysis
Source: JMIR Mhealth Uhealth. 2024 Jan 4;12:e41753. doi: 10.2196/41753 (PMC10786334; doi:10.2196/41753)

**Figure S1.** Funnel plots of study outcomes.

A: 6-minute walk test distance.


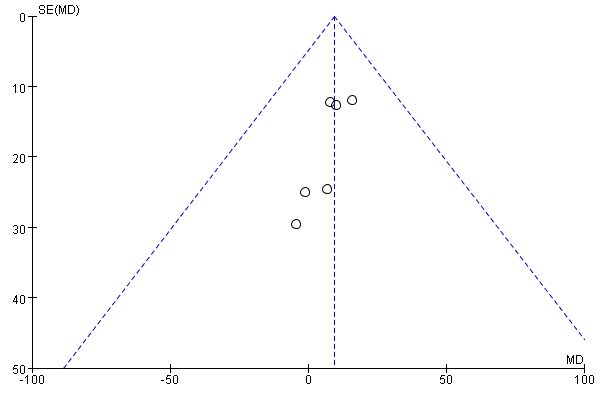


B: COPD assessment test score.


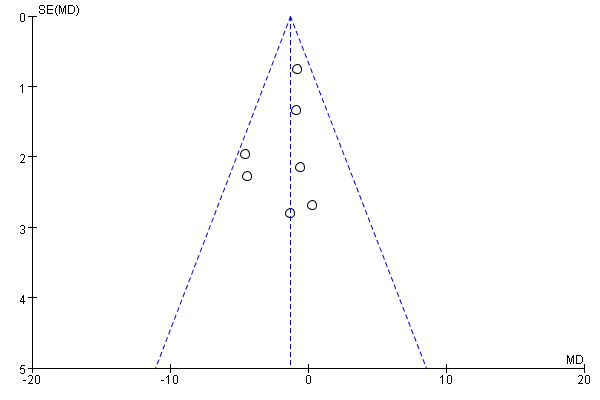


C: modified Medical Research Council dyspnea scale.


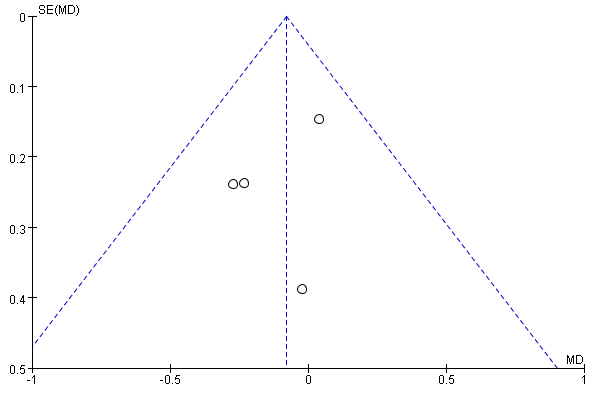


D: St. George's Respiratory Questionnaire.


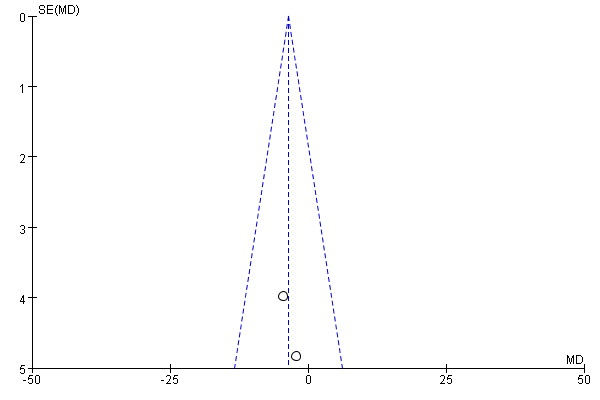


E: hospitalization from exacerbation.


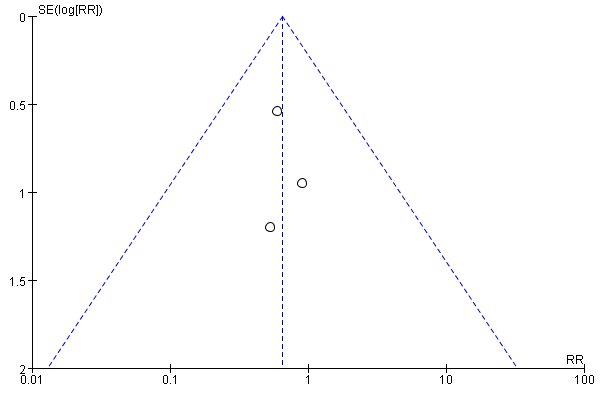


**Figure S2.** Forest plots of study outcomes between the intervention group and the control group.

Kwon (1) denotes the fixed regimen group and Kwon (2) denotes the fixed-interactive regimen group.

A: baseline 6-minute walk test distance ≥ 400 m.


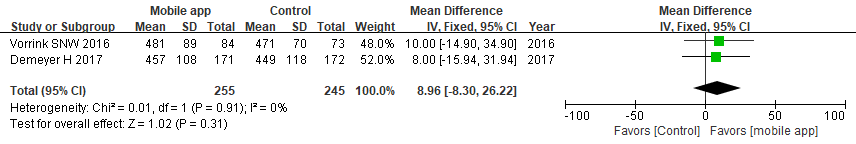


B: baseline 6-minute walk test distance < 400 m.


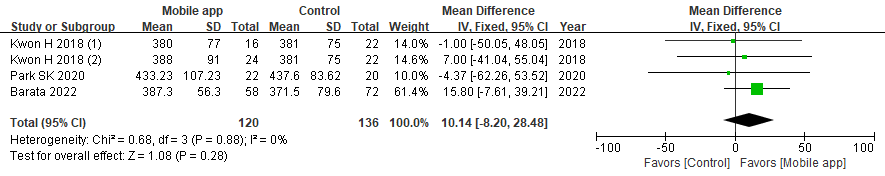


C: baseline COPD assessment test score ≥ 20.


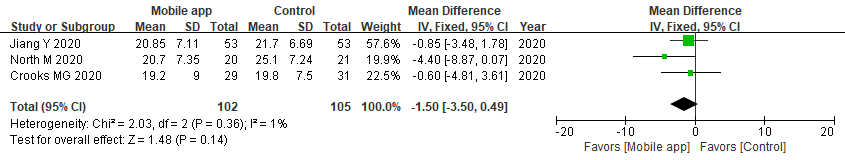


D: baseline COPD assessment test score < 20.


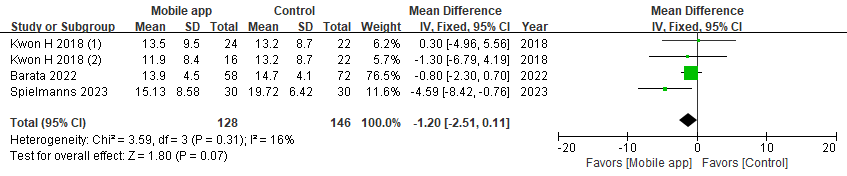


E. COPD assessment test score among studies offering exercise program only.


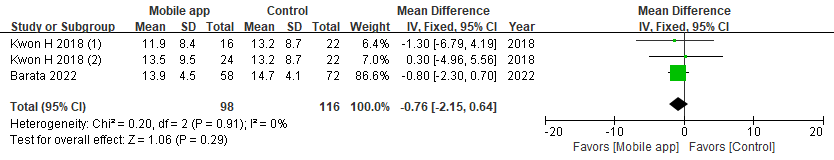


F. COPD assessment test score among studies offering both exercise and self-management program.


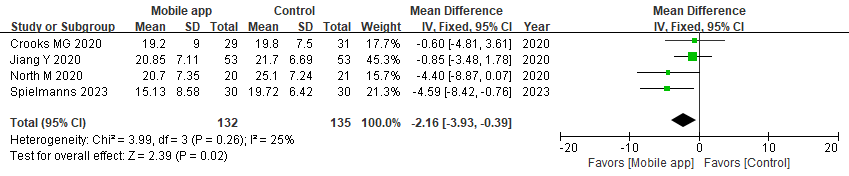

Supplement: Multimedia Appendix 3 [file mhealth-v12-e41753-s003.docx]
